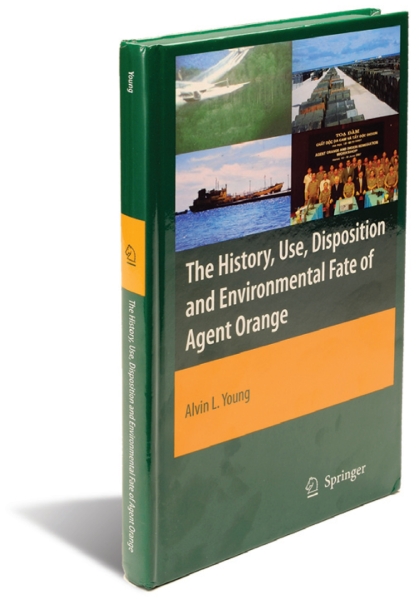# The History, Use, Disposition and Environmental Fate of Agent Orange

**Published:** 2010-06

**Authors:** Jeanne M. Stellman, Steven D. Stellman

**Affiliations:** *Jeanne M Stellman is Professor Emerita, Mailman School of Public Health, Columbia University. She was principal investigator of a major National Academy of Sciences project to develop exposure methodologies for military herbicides used in Vietnam. She is a Guggenheim fellow and Editor-in-Chief, *ILO Encyclopaedia of Occupational Health and Safety (4th ed). *She is currently a professor at SUNY Downstate Medical Center, Brooklyn, New York.*Steven D. Stellman is Professor of Clinical Epidemiology, Mailman School of Public Health, Columbia University. He holds a doctorate in physical chemistry

It has been nearly 50 years since the United States initiated a military operation that sprayed approximately 20 million gallons of phenoxy herbicides and arsenicals on South Vietnam including Agent Orange, a formulation that was contaminated with dioxin. Alvin Young has been a major player in measuring and monitoring these herbicides since the 1960s. Retired from the Air Force (AF) and now receiving support from the herbicide manufacturers Dow and Monsanto, Young remains a governmental spokesman in post-conflict remediation dealings with Vietnam. His “new” book on the issue—actually, a largely unacknowledged compilation of his and others’ writing and research—contains some valuable information. This value though, must be weighed against a number of serious deficiencies in scholarship.

Young’s book contains a gold mine of data on the herbicides, some of which have been out of public view until now. But *caveat emptor*: Page after page of text and many illustrations and photographs are taken directly from Young’s prior publications without proper citation. There are also many lengthy direct quotations from other authors that appear without quotation marks or indents. Setting aside issues of copyright, the difficulty for users who wish to cite this book’s contents will be the absolute requirement of locating all references to distinguish original from reproduced text.

A second *caveat*: Many quotations are taken out of context, emphasis has been added without acknowledgement, and other authors’ work has in some cases been misinterpreted. For example, Major General John Murray—who evaluated military records aspects of the now abandoned Agent Orange Study—is quoted as seeming to urge abandonment of all military records–based studies with Young’s emphasis-added, capitalized, and boldfaced “**NOT**.” A check in the source material, though, will show that Murray’s very next sentence reads “It is, of course, understood that eight (8) other studies which require determinations of the likelihood of Agent Orange exposure conducted by the Veterans Administration and for which the Joint Services Environmental Support Group will provide exposure determinations and military record abstractions will rigorously continue” (Murray JE. 1986. Report to the White House Agent Orange Working Group Science Subpanel on Exposure Assessment. Washington DC:Office of Science and Technology Policy). Palmer’s work is cited as supporting the notion that science cannot contribute to understanding health effects of Agent Orange, because there are too many “quality of life issues.” A check of source material (Palmer MG. 2005. The Legacy of Agent Orange: Empirical Evidence from Central Vietnam. Soc Sci Med 60:1061–1070) reveals that Palmer did not say this; further, the year of his paper is incorrectly cited.

More recently, Young has proposed a new theory to support his contention that no U.S. troops were directly sprayed upon. He asserts that because there were no reports of U.S. casualties resulting from attacks by the fighter planes that escorted the spray planes, no troops could have been in harm’s way, and he cites Flanagan, a retired AF pilot who wrote of his varied experiences in Vietnam. In fact, Flanagan’s description of a spray mission disparages the Army recordkeeping on which Young relies: “Failure was never recognized … friendly casualties could be failures, but they, too, were never identified” (Flanagan JF. 1992. Vietnam Above The Treetops: A Forward Air Controller Reports. New York:Praeger).

In the interest of full disclosure, Young describes our methodology for updating and correcting herbicide spray records as demonstrating a “woeful lack of understanding” of AF reporting procedures. We must point out in response that our work (Stellman JM, Stellman SD. 2003. Characterizing Exposure of Veterans to Agent Orange and Other Herbicides Used in Vietnam: Final Report. Washington, DC:National Academies Press) was performed in collaboration with the persons at the Department of Defense (DoD) who were the most knowledgeable about these procedures—the Center for Research on Unit Records—and co-authored by Col. Christian (retired), former director of the DoD Joint Services Environmental Support Group. Young discounts the ability of spray plane navigators to know where they were or pilots to accurately spray their targets—thereby dismissing the utility of the voluminous spray records available, despite much evidence to the contrary.

Some assertions in the book either are disingenuous or contradict Young’s earlier work. Young rationalizes in this book that the omission of maximum dioxin contaminant levels from the military specifications for herbicide quality occurred because not enough was known and measurement was difficult; but two decades ago his edited volume stated that “the manufacturers of trichlorophenol and of 2,4,5-T had been aware for many years that this class of compounds and particularly their impurities produced a toxic reaction in humans … the same manufacturers had developed, as early as 1941, a … bioassay … to monitor the production of the herbicide” (Young AL, Reggiani GM. 1988. Agent Orange and Its Associated Dioxin: Assessment of a Controversy. New York:Elsevier). Indeed, in 1965 Dow Chemical Company’s V.K. Rowe convened a highly unusual meeting of his toxicology counterparts from competitor companies to warn that the herbicide was dangerous and that processes needed cleaning up. Dow closed down and rebuilt its own feedstock operations to manage the problem. The military specifications never once mentioned dioxin for the duration of the war.

Science also seems to have ended in the 1970s and early 1980s for Young. Johnston Island, the final resting place for Agent Orange before its incineration at sea, had no important problems in 1978 according to Young. However, a 1986 AF reassessment found marine biota with up to 472 ppb dioxin, subsoil with 510 ppb dioxin, and 682,247 ppb of 2,4,5-T (Huse G, et al. 1991. Preliminary Public Health, Environmental Risk, and Data Requirements Assessment for the Herbicide Orange Storage Site at Johnston Island. Brooks Air Force Base, TX:Occupational and Environmental Health Directorate).

Finally, how very much beside the point is the book’s quibbling about whether spray estimates differ by a few percent? Here we are in 2010, some 38 years since the last spray mission overflew hamlets and fields of our ally, the South Vietnamese, and we still cannot answer these questions: Were there health consequences? Just how many “hot spots” are there? Were veterans who served in heavily sprayed areas more likely to contract illnesses than those who did not? Are there adverse effects among the people of Gulfport, Mississippi, through which millions of gallons passed, too much of it in leaky barrels? The Institute of Medicine, the editors of *Nature*, Congress, and many others have repeatedly requested that studies be done—to no avail.

These are profoundly important questions still awaiting an answer. Young contends that science is being “filtered” through the public’s confused perceptions. But while some confusion may exist, the predominant sentiment is dismay. If there is one thing that this compendium of reprinted materials shows us, it is that there is a wealth of data that can be used to answer the many open environmental questions.

## Figures and Tables

**Figure:**